# Effect of Mobilization with Movement on Pain, Disability, and Range of Motion in Patients with Shoulder Pain and Movement Impairment: A Systematic Review and Meta-Analysis

**DOI:** 10.3390/jcm12237416

**Published:** 2023-11-29

**Authors:** Daniela Dias, Mansueto Gomes Neto, Stephane da Silva Ribeiro Sales, Bárbara dos Santos Cavalcante, Palmiro Torrierri, Leonardo Roever, Roberto Paulo Correia de Araújo

**Affiliations:** 1 Physiotherapy Department, Multidisciplinary Institute of Rehabilitation and Health, Federal University of Bahia (UFBA), Salvador 40110-170, Brazil; dgarzedin@ufba.br; 2 Postgraduate Program in Interactive Processes of Organs and Systems, Federal University of Bahia (UFBA), Salvador 40170-110, Brazil; rpcaraujo@hotmail.com; 3 Physiotherapy Research Group, Physiotherapy Department, Federal University of Bahia (UFBA), Salvador 40210-905, Brazil; stephane_r.sales@hotmail.com (S.d.S.R.S.); barbaradossantoscavalcante@gmail.com (B.d.S.C.);; 4 Department of Clinical Research, Brazilian Evidence-Based Health Network, Uberlândia 38408-100, Brazil; 5 Gilbert and Rose-Marie Chagoury School of Medicine, Lebanese American University, Beirut 1401, Lebanon; 6 Biochemistry Department, Federal University of Bahia (UFBA), Salvador 40170-110, Brazil

**Keywords:** shoulder pain, disability, mobilization, meta-analysis

## Abstract

Background: Shoulder pain is a disabling musculoskeletal disorder worldwide. Thus, it is important to identify interventions able to improve pain and disability. Objective: To investigate the effects of mobilization with movement (MWM) on pain, disability, and range of motion in patients with shoulder pain and movement impairment. Methods: A systematic search of different databases was performed. The systematic review protocol has been registered in PROSPERO (CRD42023404128). A random-effects model for meta-analysis was used to determine the mean difference (MD), standardized mean differences (SMD), and 95% confidence interval for the outcome of interest. Results: Twenty-six studies were included. Of these, eighteen were included in the meta-analysis. MWM improved pain during movement with a moderate effect SMD of (−0.6; 95% confidence interval, −1.1 to −0.1, I^2^ = 0%; N = 66;) and shoulder abduction MD of (12.7°; 1.3 to 24.0; I^2^ = 73%; N = 90) compared to sham MWM in the short term (0–6 weeks). Combined MWM and conventional rehabilitation improved pain at rest, with a MD of (−1.2; −2.2 to −0.2; I^2^ = 61%; N = 100), and disability SMD of (−1.3; confidence interval −2.2 to −0.4; I^2^ = 87%; N = 185) compared to conventional rehabilitation alone in the short term. Combined MWM and conventional rehabilitation also resulted in improvement in shoulder abduction and external rotation. Compared to Maitland, MWM resulted in improvement in the shoulder abduction MD (20.4°; confidence interval 4.3 to 36.5; I^2^ = 89%; N = 130) in the short term. There is no information regarding long-term effects. Conclusion: Evidence suggests that MWM may reduce shoulder pain and restore shoulder range of motion and function. Our findings are promising, but the evidence is not strong enough to recommend it pragmatically.

## 1. Introduction

Shoulder pain with a consequent reduction in range of motion is a multifactorial disorder with a high prevalence rate [1]. Physical factors, such as lifting heavy loads, repetitive movements in awkward positions, and vibrations, are important factors in the development of shoulder pain [2]. The most frequent reason for shoulder pain is rotator cuff tendon impingement under the shoulder’s bony region [2,3]. Thus, in most patients, shoulder pain has a neuromusculoskeletal cause, with rotator cuff disorders, acromioclavicular joint disease, and glenohumeral joint disorders being the three most common causes of shoulder pain [3,4].

Shoulder pain causes pain, disability, and participation restriction. Typical primary treatment options include the use of analgesics, manual therapy, and exercises [5].

Both manual therapy and exercise programs are widely used physical therapy resources for the rehabilitation of the shoulder that presents with pain and disability [6]. Studies have demonstrated the effects of manual therapy in the management of individuals with shoulder pain [7]. Mobilization with movement is a technique that simultaneously combines the passive accessory movement of the joint with the active physiological movement of the upper and lower limbs, that is, the osteokinematic movement. The difference is that mobilization with movement is a bridge between exercises and manual therapy [8,9].

Mobilization with movement involves a set of techniques based on the theory of joint positional failure [9,10]. A sustained passive movement (glide) is applied to a painful or rigid joint, while the people perform a concomitant active movement of the joint. In the shoulder joint, a posterior and lower glide of the humeral head performed during active movements can correct the positional mechanical failure, with a consequent reduction in pain and improvement of movement [9,10]. The direction in which the auxiliary glides are administered determines how much better the formerly painful or constrained movement is now. Thus, this technique combines physiological and accessory joint movements, has been proposed as a manual therapy technique to improve joint range of motion and reduce pain [8,9], and is widely used in managing musculoskeletal pain [10]. 

In a systematic review, Ho et al. [11] determined the effects of manual therapy in managing dysfunctions of the shoulder. They concluded that the evidence was conflicting for the use of manual therapy in managing unspecific shoulder pain to decrease pain and restore function in the short term compared to other interventions. However, further studies are required to confirm these results [11]. Recently, Stathopoulos et al. conducted a systematic review [12] and concluded that compared to a sham mobilization, passive, or no therapeutic approach, mobilization with movement produced a clinically significant increase in the short term in the range of motion in shoulder adhesive capsulitis and hip pain. Since the previous review was published [10,11,12], randomized controlled trials have been completed. Additionally, to our knowledge, no meta-analysis has been performed on mobilization with movement for the treatment of shoulder dysfunction (pain and movement impairment) and/or comparing different mobilization techniques. 

Although the previous systematic review has provided important information on the effects of mobilization with movement, more up-to-date information on general and specific effects and the quality of evidence in approaching individuals with musculoskeletal disorders in the shoulder is needed. In addition, this systematic review with meta-analysis may provide information about the prescription of the technique in relation to the number of sessions, frequency, and duration of effects, which are not yet well established in the literature.

Thus, we performed this systematic review and meta-analysis to analyze published randomized controlled trials (RCTs) on the effects of mobilization with movement in clinically relevant outcomes, such as pain, disability, and range of motion in people with shoulder pain and movement impairment.

## 2. Methods

The Preferred Reporting Items for Systematic Reviews and Meta-Analyses (PRISMA) guidelines [13] was used to report this systematic review. This study was registered in the PROSPERO (CRD42023404128).

RCTs were qualified if they satisfied the following PICOS requirements: (a) population, included adult patients (aged ≥ 18 years) with shoulder dysfunction (pain and/or movement impairment) independent of time or cause; (b) intervention, mobilization with movement applied to short-term (0–6 weeks) or long-term (≥6 weeks); (c) comparator, other intervention (other types of joint mobilization, sham mobilization, exercise, etc.), or control (no exercise); (d) Outcomes, pain, disability, range of motion, and/or function; (e) study design, a randomized controlled clinical trial. The primary outcomes of interest were pain (measured using a visual analog scale, numerical rating scale, or other valid and reliable instrument), range of motion, disability and/or function assessed with valid and reliable questionnaires. In this review, mobilization with movement was considered as the prescribed manual therapy involving manual application of a sustained glide by a therapist to a joint while a concurrent movement of the joint is actively performed by the patient. A sustained glide is applied to a painful shoulder joint, while the people performs a concomitant and active movement of the shoulder [8,9].

We screened the MEDLINE/PubMed, PEDro Database, Scientific Electronic Library Online and the Cochrane Central up to June 2023 without language restrictions or publication status restrictions for relevant studies. We used a standard protocol for this search with a controlled vocabulary (mesh term for MEDLINE/PubMed and Cochrane). Study design, participants, and interventions were the three categories of terms and their synonyms that we employed in our search approach. Two reviewers independently conducted the search.

The strategy developed by Higgins and Green [14] was used to identify studies in MEDLINE/PubMed and Cochrane Central. The search strategy for MEDLINE/PubMed is presented in Supplementary Material Table S1. A search strategy using similar terms was used to identify randomized controlled trials from other databases. We checked the list of references of the articles to identify other potentially eligible studies. 

Two reviewers independently assessed each data source’s collection of titles and abstracts. The full text was requested for a thorough evaluation if at least one of the reviewers thought that one of them was eligible. The full texts of the chosen papers were then independently evaluated by the two reviewers to determine whether they satisfied the requirements for inclusion or exclusion. Any disagreements were resolved by a third reviewer. We also checked each selected article’s reference list to identify other potentially eligible studies. Two authors independently extracted data from the published reports by using standard data extraction forms adapted from Cochrane Collaboration [14]. Any disagreements were resolved by a third reviewer. Aspects of the study population, intervention performed, follow-up period, rates of missing data, outcome measures, and results were reviewed. 

The methodological quality of RCTs included was scored by two researchers by using the Physiotherapy Evidence Database scale [15]. The scale consists of 10 criteria, each receiving either a yes or no score. The Physiotherapy Evidence Database scale is a useful tool for assessing the quality of rehabilitation RCTs [15,16,17]. Any disagreements in the rating of the studies were resolved by a third reviewer. Physiotherapy Evidence Database scale scores of 0–3 were considered ‘poor’, 4–5 ‘fair’, 6–8 ‘high’, and 9–10 ‘excellent’.

The weighted mean difference between groups was used to express the pooled-effect estimates, which were calculated by comparing the least square means of change from baseline to endpoint for each group. Results for continuous variables were presented as the mean difference (MD) between the randomized groups’ changes in the variable. Based on recently developed techniques, nonparametric data were converted to means and standard deviation (SD) [18]. When the SD of change was not available, but the confidence interval (CI) was available, we converted the CI to SD according to the method recommended by Cochrane Collaboration [14]. Calculations were performed using a random-effects model. 

Four comparisons were made: (1) mobilization with movement versus sham mobilization with movement, (2) combined mobilization with movement and conventional rehabilitation versus conventional rehabilitation alone, (3) mobilization with movement versus end range mobilization, and (4) mobilization with movement versus exercise. Calculations were made for mean differences, standardized mean differences, and 95% CI. When various instruments or measures were utilized in the studies to examine the same outcome, a standardized mean difference (SMD) was used in the meta-analysis. A SMD of 0.2, 0.5, and 0.8 was interpreted as a small, moderate, and large effect size, respectively [14]. For the outcome pain, we consider a mean difference above 2 points on a scale of 0 to 10 as clinically significant. Statistical significance was set at a value of 0.05. Heterogeneity among studies was examined using I^2^ statistic and Cochran’s Q, in which values >40% were considered indicative of high heterogeneity [16]. Meta-analyses were performed using the Review Manager (Version 5.4) [19]. We used Cohen’s kappa to assess agreement between reviewers in the selection of studies.

The certainty of evidence on primary outcomes was assessed using the Grading of Recommendations Assessment, Development, and Evaluation (GRADE) approach. GRADEpro GDT 2015 was used to import data from Review Manager to create a “Summary of findings table”. Risk of bias, precision of estimates, consistency, indirectness, and publishing bias were the five components of the assessment [14]. We performed downgrading by one level for risk of bias when more 1/4 of the studies included in the pooled estimate were considered at a high risk of bias. Results were considered imprecise if the total sample size was <300 and <400 for dichotomous and continuous outcomes, respectively. We performed downgrading by one level if the heterogeneity between RCTs was substantial (ie. I^2^ > 40%). Publishing bias was evaluated by visual inspection of funnel plots (a scatterplot of the ES from individual studies against its SE). When appropriate, footnotes and comments were used to support decisions to downgrade the certainty of studies.

## 3. Results

**Figure 1:**
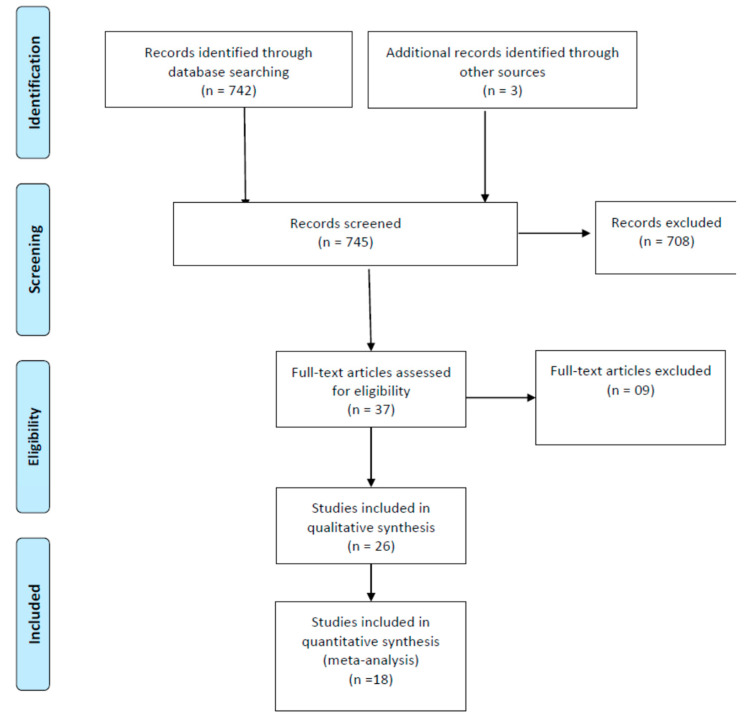
Flow diagram showing the reference screening and study selection.

A total of 692 abstracts were found in the initial search, and 32 potentially pertinent studies were retrieved for a more thorough examination. After reading all 32 articles, 6 were removed. The agreement between the reviewers, assessed with the Cohen’s kappa, was 0.88. Finally, 26 papers [20,21,22,23,24,25,26,27,28,29,30,31,32,33,34,35,36,37,38,39,40,41,42,43,44,45] met the eligibility criteria following PICOS criteria, Table S2, (Supplementary Material Table S2). Figure 1 is the PRISMA flow diagram of RCTs included. The studies were scored using the Physiotherapy Evidence Database scale. Table S2 also presents the results of individual assessment by using the Physiotherapy Evidence Database scale (Supplementary Material Tables S2–S6). The random allocation sequence’s generation and concealment processes were poorly described. Six investigations provided unbiased proof of the characteristics of random allocation and the balance of baseline characteristics. Only three studies claimed to have used blinded measures. 

Of the 26 randomized controlled trials included in this review, 7 studies compared the combination of mobilization with movement and conventional rehabilitation to conventional rehabilitation, 4 studies compared mobilization with movement to sham mobilization with movement, 4 studies compared mobilization with movement to end range mobilization, 1 study compared mobilization with movement to end range mobilization and passive sustained joint mobilization, 1 study compared combined mobilization with movement and conventional rehabilitation to combined end range mobilization and conventional rehabilitation, 2 studies compared mobilization with movement to exercise alone, 2 studies compared mobilization with movement to passive sustained joint mobilization, 1 study compared mobilization with movement with positional release therapy, 1 study compared combined mobilization with movement and kinesio-taping to mobilization with movement alone, 1 study compared combined mobilization with movement and kinesio-taping to exercise alone, 1 study compared mobilization with movement with cryotherapy, and 1 study compared mobilization with movement to muscle energy technique.

The number of people in the RCTs included ranged from 20^30^ to 100^33^. Mean age ranged from 31.6 to 83.9 years. All studies analyzed in this review included outpatients with documented shoulder dysfunction. Table S1 summarizes the characteristics of the included studies.

The variables that were applied when mobilization with movement was employed have been documented. The duration of mobilization with movement programs ranged from 1 to 8 weeks. The number of sessions each week varied from 1 to 7. The mobilization with movement intervention characteristics in the included studies are provided in Table 1.

### 3.1. Mobilization with Movement vs. Sham Mobilization with Movement

**Figure 2:**
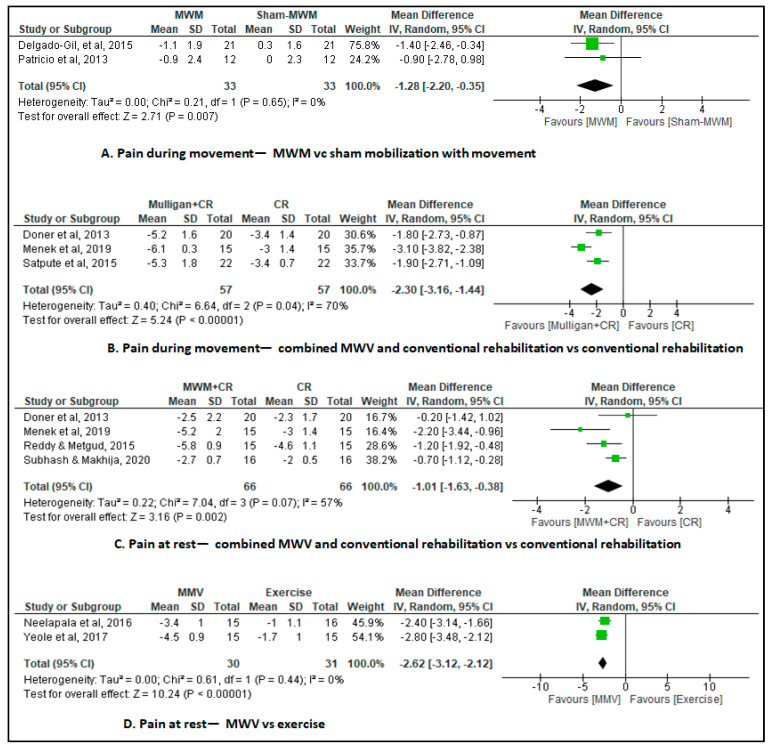
Changes in pain [23,24,28,31,32,33,35,38,39].

Two studies [26,33] assessed pain during movement as the outcome (with a visual analog scale/numerical rating scale 0–10 cm). The mobilization with movement group and the sham mobilization with movement group each comprised 33 patients. The results showed a reduction in pain in the short term (0–6 weeks) with a moderate effect size (MD of −1.28, 95% CI, −2.2 to −0.4; I^2^ = 0%; 2 studies, N = 66; low-certainty evidence, downgraded for risk of bias and imprecision) during activity in the mobilization with movement group participants versus the sham mobilization with movement group participants (Figure 2A).

#### Combined Mobilization with Movement and Conventional Rehabilitation versus Conventional Rehabilitation

Three studies [20,29,32] assessed pain during movement as the outcome (with a visual analog scale 0–10 cm). The combined mobilization with movement and conventional rehabilitation group and the conventional rehabilitation group each comprised 57 patients. The meta-analysis showed a reduction in pain in the short-term MD of −2.3 cm (95% CI, −3.2 to −1.4; I^2^ = 70%; 3 studies, N = 114; very low-certainty evidence, downgraded for risk of bias, inconsistency, and imprecision) during activity in the combined mobilization with movement and conventional rehabilitation group participants versus the conventional rehabilitation group participants (Figure 2B). As the MD was greater than 2 points, it can be considered clinically significant and relevant.

### 3.2. Combined Mobilization with Movement and Conventional Rehabilitation vs. Conventional Rehabilitation

Three studies [20,27,32,40] assessed pain at rest as the outcome (with a visual analog scale 0–10 cm). The combined mobilization with movement and conventional rehabilitation group and the conventional rehabilitation group each comprised 66 participants. The meta-analysis showed a reduction in pain in the short-term MD of −1.0 cm (95% CI, −1.6 to −0.4; I^2^ = 57%; 4 studies, N = 132; very low-certainty evidence, downgraded for risk of bias, inconsistency, and imprecision) at rest in the combined mobilization with movement and conventional rehabilitation group participants versus the conventional rehabilitation group participants (Figure 2C).

### 3.3. Mobilization with Movement vs. Exercise

Two studies [22,24] assessed pain at rest as the outcome (with a visual analog scale 0–10 cm). The mobilization with movement group comprised 30 patients and the exercise group comprised 31 patients. The meta-analysis showed a reduction in pain in the short-term MD of −2.6 cm (95% CI, −3.1 to −2.1; I^2^ = 61%; 2 studies, N = 61; low-certainty evidence, downgraded for risk of bias and imprecision) in the mobilization with movement group participants versus the exercise group participants (Figure 2D). As the MD was greater than 2 points, it can be considered clinically significant and relevant.

### 3.4. Combined Mobilization with Movement and Conventional Rehabilitation vs. Conventional Rehabilitation

**Figure 3:**
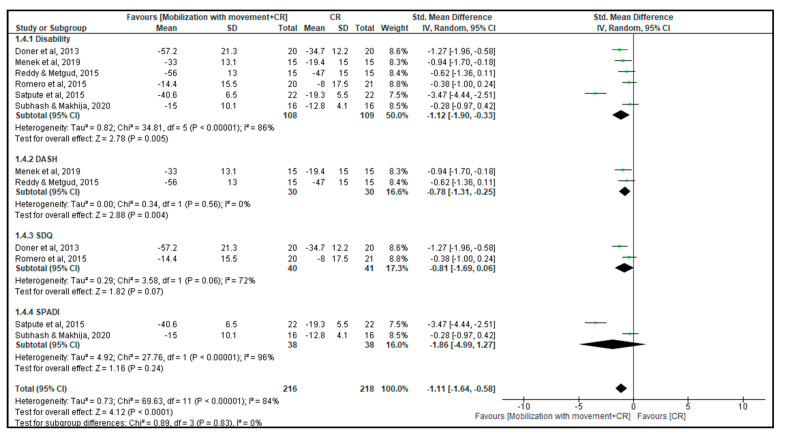
Change in disability [23,24,33,34,35,38].

Six studies assessed disability [20,27,28,29,32,40]. Two studies measured disability using the Disabilities of the Arm, Shoulder, and Hand Score [20,27], two using the Strengths Difficulties Questionnaire [28,32], and two using the Shoulder Pain and Disability Index I [29,40]. In four of the trials, significant improvements were found in the combined mobilization with movement and conventional rehabilitation group compared to the conventional rehabilitation group. Because different instruments were used to assess the disability, we performed the meta-analysis using the SMD. The results showed an improvement in disability in the short-term, with a large effect size (SMD of −1.12, 95% CI: −1.9 to −0.3, I^2^ = 83%; 6 studies, N = 217; very low-certainty evidence, downgraded for risk of bias, inconsistency, and imprecision) (Figure 3) in the combined mobilization with movement and conventional rehabilitation group participants compared to the conventional rehabilitation group participants.

As assessed using the Disabilities of the Arm, Shoulder and Hand Score, a significant difference in disability standardized mean difference of −0.8 (95% CI: −1.3 to −0.3, N = 60; Figure 3) was noted in the combined mobilization with movement and conventional rehabilitation group participants compared to the conventional rehabilitation group participants. As assessed using the Strengths Difficulties Questionnaire, a non-significant difference in disability with a large effect size (SMD of −0.8, 95% CI: −1.7 to 0.1, N = 81; Figure 3) was noted in the combined mobilization with movement and conventional rehabilitation group participants compared to the conventional rehabilitation group participants. As assessed using the Shoulder Pain and Disability Index, no difference in disability with a large effect size (standardized mean difference of −1.9, 95% CI: −5.0 to 1.3, N = 72; Figure 3) was noted in the combined mobilization with movement and conventional rehabilitation group participants compared to the conventional rehabilitation group participants.

### 3.5. Mobilization with Movement vs. Sham Mobilization with Movement

**Figure 4:**
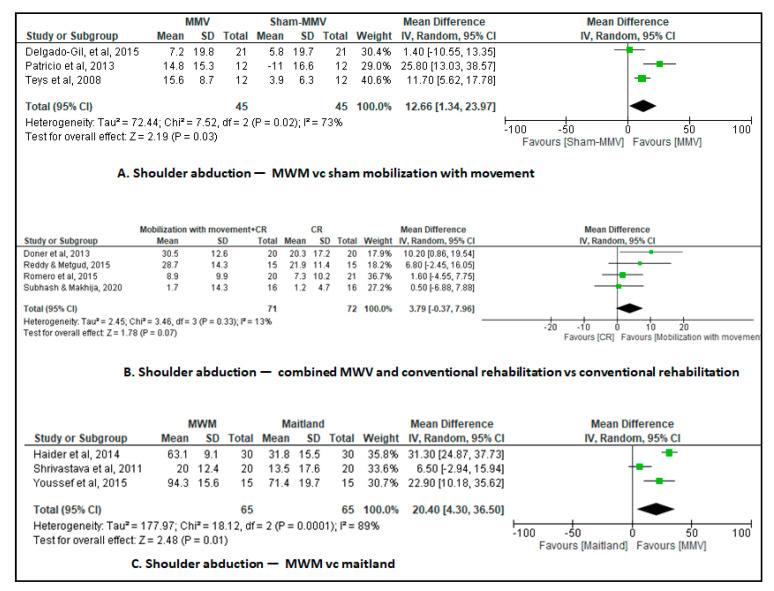
Changes in shoulder abduction [23,32,33,34,36,37,38,39,42,44].

Three studies [26,33,38] assessed active abduction range of motion of the shoulder as the outcome. The mobilization with movement group and the sham mobilization with movement group each comprised 45 patients. The meta-analysis showed an improvement in shoulder abduction in the short-term MD of 12.7° (95% CI, 1.3 to 24.0; I^2^ = 73%; 3 studies, N = 90; very low-certainty evidence, downgraded for risk of bias, inconsistency, and imprecision) in the mobilization with movement group participants versus the sham mobilization with movement group participants (Figure 4A).

### 3.6. Combined Mobilization with Movement and Conventional Rehabilitation vs. Conventional Rehabilitation

Four studies [27,28,32,40] assessed shoulder abduction range of motion as the outcome. The combined mobilization with movement and conventional rehabilitation group comprised 55 patients and the conventional rehabilitation group comprised 56 patients. The meta-analysis showed an improvement in shoulder abduction in the short-term MD of 13.2° (95% CI, 4.1 to 22.2; I^2^ = 67%; 4 studies, N = 143; very low-certainty evidence, downgraded for risk of bias, inconsistency, and imprecision) in the combined mobilization with movement and conventional rehabilitation group participants versus the conventional rehabilitation group participants (Figure 4B).

### 3.7. Mobilization with Movement vs. End Range Mobilization

Three studies [30,31,36] assessed shoulder abduction range of motion as the outcome. The mobilization with movement group and the end range mobilization group each comprised 65 patients. The meta-analysis showed an improvement in shoulder abduction in the short-term MD of 20.4° (95% CI, 4.3 to 36.5; I^2^ = 89%; 3 studies, N = 130; very low-certainty evidence, downgraded for risk of bias, inconsistency, and imprecision) in the mobilization with movement group participants versus the end range mobilization group participants (Figure 4C).

### 3.8. Combined Mobilization with Movement and Conventional Rehabilitation vs. Conventional Rehabilitation

**Figure 5:**
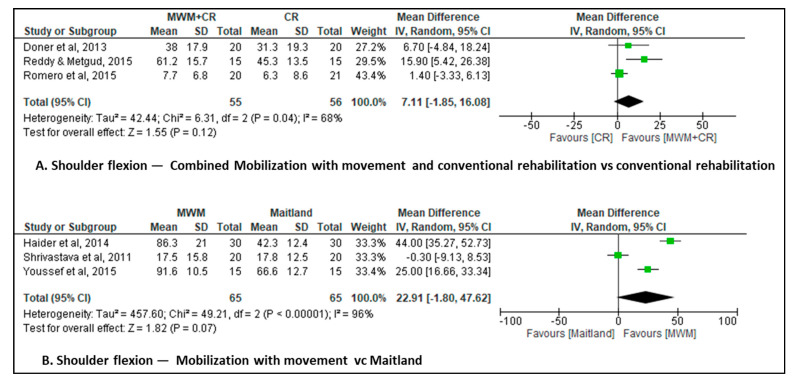
Changes in shoulder flexion [33,34,36,37,38,42].

Three studies [27,28,32] assessed shoulder flexion range of motion as the outcome. The combined mobilization with movement and conventional rehabilitation group comprised 55 patients and the conventional rehabilitation group comprised 56 patients. The meta-analysis showed no difference in shoulder flexion in the short-term MD of 4.8° (95% CI, 0.3 to 9.3; I^2^ = 20%; 3 studies, N = 111; very low-certainty evidence, downgraded for risk of bias, inconsistency, and imprecision) in the combined mobilization with movement and conventional rehabilitation group participants versus the conventional rehabilitation group participants (Figure 5A).

### 3.9. Mobilization with Movement vs. End Range Mobilization

Three studies [30,31,36] assessed shoulder flexion range of motion as the outcome. The mobilization with movement group and the end range mobilization group each comprised 65 patients. The meta-analysis showed no difference in shoulder flexion in the short-term MD of 22.9° (95% CI, −1.8 to 47.6; I^2^ = 96%; 3 studies, N = 130; very low-certainty evidence, downgraded for risk of bias, inconsistency, and imprecision) in the mobilization with movement group participants versus the end range mobilization group participants (Figure 5B).

### 3.10. Combined Mobilization with Movement and Conventional Rehabilitation vs. Conventional Rehabilitation

Four studies [27,28,32,40] assessed shoulder external rotation range of motion as the outcome. The combined mobilization with movement and conventional rehabilitation group comprised 71 patients and the conventional rehabilitation group comprised 72 patients. The meta-analysis showed no difference in shoulder external rotation in the short-term MD of 3.8° (95% CI, −0.4 to 7.9; I^2^ = 13%; 4 studies, N = 143; low-certainty evidence, downgraded for risk of bias and imprecision) in the combined mobilization with movement and conventional rehabilitation group participants versus the conventional rehabilitation group participants.

## 4. Discussion

This systematic review indicated with a very low-certainty evidence that mobilization with movement was more effective and clinically relevant than sham mobilization with movement and convention rehabilitation to reduce pain and improve the range of motion in the short-term. Although our findings are promising, the available evidence is not strong enough to recommend it pragmatically. 

Four studies demonstrated an improvement with a large effect size in disability in patients treated with combined mobilization with movement and conventional rehabilitation compared with those treated with conventional rehabilitation alone in the short-term. In addition, when compared to end range mobilization, mobilization with movement resulted in an improvement in the shoulder range of motion. Despite these findings, it is important to note that the certainty of the evidence found was low to very low, in addition to highlighting the uncertainties about the effect of the treatment.

This systematic review and meta-analysis analyzed mobilization with movement as a potential intervention in the musculoskeletal rehabilitation of patients with shoulder pain and movement impairment. Moreover, we included pain and self-reported disability measures, which are the important outcomes in musculoskeletal rehabilitation. We also compared mobilization with movement with other mobilization techniques and exercises. All these factors make this review an important one in the literature. Our results are consistent with the findings of previous studies on the effects of mobilization with movement on shoulder pain [11,12].

Regarding the outcome pain at rest, we identified an improvement of more than 20% in all the comparisons made, representing a mean difference of 1 to 4 points on a scale of 0 to 10. Regarding pain at rest, for patients with shoulder conditions, the minimal important difference for pain at rest on a visual analogue scale (0–10) should at least be 3 Regarding the outcome pain during movement, we also identified an improvement of more than 20% in all the comparisons made, representing a mean difference of 5 points on a scale of 0 to 10. Considering pain during activity, for patients with shoulder conditions, the minimal important differences for pain on a visual analog scale (0–10) must be at least 2.1 [46]. For the disability outcome, the identified effect size was greater than 0.8 and was considered a large effect size.

The duration of observations in the included trials varied from 1 to 8 weeks, and the mobilization with movement protocols included exercises at a frequency of 1–7 times per week, but the session duration was unreported in most of the studies. Because a standard protocol and definition for mobilization with movement are unestablished, there is a wide variation among studies on how the mobilization with movement was implemented. Determining the most appropriate mobilization prescription (subject position, movement, joint alignment, dosage, time, frequency, and duration) is important to achieve optimal results on self-reported outcome measures.

## 5. Limitations

Despite the findings, the absence of high-quality RCTs limits the conclusions of this systematic review. Additionally, it was established that the evidence qualities for the outcomes of pain and disability were low and very low, respectively. The included papers in this review did not properly report concealment allocation. Thus, in studies with adequate concealment allocation, the efficacy of mobilization with movement can be much lower. For patients with shoulder pain and movement impairment, a clear-cut and practical recommendation about mobilization with movement is challenging to formulate. Moreover, caution is warranted while interpreting our results because of the significant heterogeneity found in the main analysis, although this ultimately reflects the body of evidence about mobilization with movement and relevant outcomes for patients with shoulder pain and movement impairment. One of the authors of the review is Mulligan concept instructor accredited; nevertheless, this researcher did not take part in the selection, risk of bias assessment, and data analysis stages. Finally, in this review we used four databases to identify the studies, which may limit the range of studies included.

Additional studies are warranted to ascertain the beneficial effects of mobilization with movement over a period and to determine their essential attributes, such as plane of motion, positioning, manual glide movement, volume, frequency, and duration. 

## 6. Conclusions

Very low-certainty evidence suggests that mobilization with movement may reduce pain and increase shoulder range of motion and function in the short-term. The determined effect size for the disability result was larger than 0.8 and was regarded as a large effect size. Despite this, it is important to note the uncertainties about the treatment effect, due to the high heterogeneity found.

## Figures and Tables

**Table 1:** Characteristics of the mobilization with movement Intervention in the trials included in the review.

Study	Groups	Intervention	Volume (Repetition Sets)	Frequency (Times a Week)	Time (Minutes)	Duration (Week)
Chandrasekaran et al., 2021 [20]	Mobilization with movement (*n* = 15) Positional release technique (*n* = 15)	Mobilization with movement Positional release technique	NR	NR	NR	2
Rana et al., 2021 [21]	Mobilization with movement (*n* = 20) Maitland mobilization (*n* = 20)	Mobilization with movement + conventional exercises Maitland mobilization + conventional exercises	NR	NR	NR	6
Fernandes et al., 2020 [22]	Group M—Mobilization with movement (*n* = 28) Group K—Kaltenborn (*n* = 28)	Mobilization with movement Kaltenborn mobilization	3 × 10	3	NR	2
Subhash and Makhija, 2020 [23]	Mobilization with movement (*n* = 16) Control group (*n* = 16)	Mobilization with movement + Conventional physiotherapy Conventional physiotherapy	3 × 7	1	NR	2
Menek et al., 2018 [24]	Mobilization with movement (*n* = 15) Control group (*n* = 15)	Mobilization with movement + Conventional physiotherapy Conventional physiotherapy	3 × 10	3	20	NR
Ragav and Singh, 2019 [25]	Group A—Mobilization with movement (*n* = 10) Group B—Kaltenborn (*n* = 10) Control C—Control group (*n* = 10)	Mobilization with movement Kaltenborn mobilization Hot water fomentation and home-based range of motion exercises program of the shoulder joint	3 × 10	6	NR	3
Rayudu and Alagingi, 2018 [26]	Mobilization with movement (*n* = 30) Control group (*n* = 30)	Mobilization with movement + Conventional exercises Muscle energy technique + conventional exercises	3 × 10	3	NR	3
Srivastava et al., 2018 [27]	Mobilization with movement (*n* = 11) Cryotherapy (*n* = 11)	Mobilization with movement + impairment-based exercises Cryotherapy + impairment-based exercises	3 × 10	1	NR	1
Yeole et al., 2017 [28]	Mobilization with movement (*n* = 15) Control group (*n* = 15)	Mobilization with movement + Supervised exercises Supervised exercises	NR	1	NR	1
Neelapala et al., 2016 [31]	Mobilization with movement (*n* = 15) Control group (15)	Mobilization with movement Supervised exercises	NR	3	NR	1
Guimarães et al. 2016 [29]	Mobilization with movement (*n* = 14) Control group (*n* = 13)	Mobilization with movement + Sham Sham + mobilization with movement	3 × 10	2	NR	3
Delgado Gil et al. 2015 [32]	Mobilization with movement (*n* = 21) Control group (*n* = 21)	Mobilization with movement Sham	3 × 10	2	10	2
Romero et al. 2015 [34]	Mobilization with movement (*n* = 22) Control group (*n* = 22)	Mobilization with movement + standard physiotherapy Standard physiotherapy	3 × 10	3	20	2
Satpute et al. 2015 [35]	Mobilization with movement (*n* = 22) Control group (*n* = 22)	Mobilization with movement + exercise + hot pack Exercise + hot pack	3 × 10	3	NR	3
Reddy and Metgud, 2015 [33]	Mobilization with movement (*n* = 15) Control group (*n* = 15)	Mobilization with movement + conventional physiotherapy Conventional physiotherapy	3 × 10	15 sessions followed	NR	2
Youssef et al., 2015 [36]	Mobilization with movement (*n* = 15) Control group (*n* = 15)	Mobilization with movement + conventional physiotherapy Conventional Physiotherapy	3 × 10	3	NR	6
Haider et al., 2014 [37]	Mobilization with movement (*n* = 60) Control group (*n* = 60)	Mobilization with movement Maitland’s technique	NR	NR	30	8
Arshad et al., 2015 [30]	Mobilization with movement (*n* = 50) Control group (*n* = 50)	Mobilization with movement + Ultrasound + transcutaneus electrical nerve stimulation + home plan for exercises Maitland + ultrasound + transcutaneus electrical nerve stimulation + home plan for exercises	NR	2	NR	8
Doner et al. 2013 [38]	Mobilization with movement (*n* = 20) Control group (*n* = 20)	Hot pack + transcutaneus electrical nerve stimulation + mobilization with movement Hot pack + transcutaneus electrical nerve stimulation + stretching	3 × 10	5	NR	3
Patrício et al. 2013 [39]	Mobilization with movement (*n* = 12) Control group (*n* = 12)	Mobilization with movement Placebo	3 × 10	2	30	3
Teys et al. 2013 [40]	Mobilization with movement (*n* = 13) Control group (*n* = 12)	Mobilization with movement Mobilization with movement + tape	3 × 10	1	NR	2
Djordjevic et al., 2012 [41]	Mobilization with movement (*n* = 10) Control group (*n* = 10)	Mobilization with movement + Kinesio-taping Supervised exercises	3 × 10	5	NR	2
Shrivastava et al. 2011 [42]	Mobilization with movement (*n* = 20) Grupo Intervenção (*n* = 20)	Mobilization with movement + hot pack + exercises Maitland + hot pack + exercises	NR	6	NR	2
Kachingwe et al., 2008 [43]	Exercises (*n* = 8) Exercises + Manual mobilization (*n* = 9) Mobilization with movement (*n* = 9) Control group (*n* = 7)	Supervised exercises Supervised exercises + glenoumeral mobilization; Supervisioned exercises + mobilization with movement Control group with orientations	3 × 10	NR	NR	NR
Teys et al. 2008 [44]	Mobilization with movement (*n* = 12) Control group (*n* = 12)	Mobilization with movement Sham mobilization with movement	3 × 10	3	NR	1
Yang et al. 2007 [45]	Mobilization with movement (*n* = 14) Control group (*n* = 14)	Mobilization with movement Middle and end range mobilization	3 × 10	2	30	12

NR—not reported.

## Data Availability

The authors will freely share the unfiltered raw data that underlie the results of this article.

## Supplementary Materials

The following supporting information can be downloaded at: https://www.mdpi.com/article/10.3390/jcm12237416/s1, Table S1: Search strategy Pubmed and Cochrane data base; Table S2: Characteristics of the included studies and PEDro Score [20,21,22,23,24,25,26,28,29,30,31,32,33,34,35,36,37,38,39,40,41,42,43,44,45]; Table S3: Summary of findings; Table S4: Summary of findings; Table S5: Summary of findings; Table S6: Summary of findings.
