# Supplementary material for: Effect of Mobilization with Movement on Pain, Disability, and Range of Motion in Patients with Shoulder Pain and Movement Impairment: A Systematic Review and Meta-Analysis
Source: J Clin Med. 2023 Nov 29;12(23):7416. doi: 10.3390/jcm12237416 (PMC10706990; doi:10.3390/jcm12237416)
Supplement: Supplementary file 1 [file jcm-12-07416-s001.zip › jcm-2581653-supplementary.pdf]

Supplementary Material

Table S1: Search strategy Pubmed and Cochrane data base.

1. ((((((("Shoulder"[Mesh]) OR "Shoulder Pain"[Mesh]) OR "Shoulder Impingement Syndrome"[Mesh]) OR "Shoulder Joint"[Mesh])) OR (("Shoulder Pain"[Mesh]) OR ((Pain, Shoulder) OR (Pains, Shoulder) OR (Shoulder Pains)))))

2. ((((((((((((randomized controlled trial) OR Random Allocation) OR Controlled Clinical Trials) OR Control groups) OR (Clinical trials/ OR clinical trials, phase i/ OR clinical trials, phase ii/ OR clinical trials, phase iii/ OR clinical trials, phase iv)) OR Clinical Trial) OR Clinical Trials Data Monitoring Committees) OR Double-blind method) OR Single-blind method) OR Placebos) OR Placebo effect) OR Cross-over studies) OR Multicenter Studies))

3. (((mobilization with movement) OR mulligan mobilization) OR (("Musculoskeletal Manipulations"[Mesh]) OR ((Manipulations, Musculoskeletal) OR (Manual Therapies) OR (Manual Therapy) OR (Therapies, Manual) OR (Therapy, Manual) OR (Manipulation Therapy) OR (Manipulation Therapies) OR (Therapies, Manipulation) OR (Manipulative Therapies) OR (Manipulative Therapy) OR (Therapies, Manipulative) OR (Therapy, Manipulative) OR (Therapy, Manipulation))))

4. 1 AND 2 AND 3

Table S2: Characteristics of the included studies and PEDro Score.

Study	Population			Outcome measures	Adverse Effects	Dropout (%)	Pedro score
	Condition	Sample Size<br>Age (year)	Gender (M/F)				
Chandrasekaran et al, 2021 [1]	Adhesive capsulitis	30<br>NR	NR	Range of Motion (Goniometer)<br>Disability (Shoulder Pain Disability Index)	NR	4	4
Rana et al, 2021 [2]	Adhesive capsulitis	40<br>53	4;16	Range of Motion (Goniometer)<br>Disability (Shoulder Pain Disability Index)	NR	NR	4
Fernandes et al, 2020 [3]	Adhesive capsulitis	56<br>NR	33/23	Range of Motion (Goniometer)<br>Disability (Shoulder Pain Disability Index)	NR	4	4
Subhash & Makhija, 2020 [4]	Shoulder dysfunction	32<br>31,6y	NR	Pain intensity (Numeric pain rating scale)<br>Pain free shoulder range of motion in flexion, abduction, internal rotation and external rotation (Goniometer)<br>Disability (Shoulder Pain Disability Index)	NR	NR	4
Ragav & Singh, 2019 [5]	Adhesive capsulitis	30<br>NR	19/11	Pain (Visual Analogue Scale)<br>Range of Motion (Goniometer)<br>Disability (Shoulder Pain Disability Index)<br>Quality of Life (Short Form 36)	NR	NR	6
Menek et al, 2019 [6]	SIS	30<br>50,9y	18/12	Pain (Visual Analogue Scale, Neer Shoulder Test and Hawkins-Kennedy Shoulder Test)<br>Range of Motion (Goniometer)	NR	NR	5

				Disability (Disabilities of the Arm Shoulder and Hand)<br>Quality of Life (Short Form 36)			
Rayudu & Alagingi, 2018 [7]	Adhesive capsulitis	60<br>NR		Pain and Function (Shoulder Pain Disability Index)<br>Range of motion (Goniometer)	NR	NR	6
Yeole et al, 2017 [8]	Adhesive capsulitis	30<br>56,3y	16/14	Range of Motion (Goniometer)<br>Disability (Shoulder Pain Disability Index)	NR	NR	4
Neelapala et al, 2016 [9]	Shoulder pain	31<br>41,3y	12/11	Pain (Visual Analogue Scale)<br>Shoulder rotator strength (external e internal rotation)<br>Scapular motor control Upward rotation	NR	4(13)	8
Guimarães et al. 2016 [10]	SIS	27<br>31y	10/15	Range of Motion (Goniometer)<br>Disability (Shoulder Pain Disability Index / Disabilities of the Arm Shoulder and Hand)	NR	NR	8
Delgado Gil et al. 2015 [11]	SIS	42<br>55y	8/34	Pain (Visual Analogue Scale)<br>Range of Motion (Goniometer)	NR	NR	7
Romero et al. 2015 [12]	Shoulder dysfunction	44<br>83,9 y	22/22	Pain (Visual Analogue Scale)<br>Range of Motion (Goniometer)<br>Disability (S-Strengths and Difficulties Questionnaire)	NR	3(6,8)	7
Satpute et al. 2015 [13]	Shoulder pain	44(41)<br>53 y	25/19	Pain (Visual Analogue Scale)<br>Range of Motion (Goniometer)<br>Disability (Shoulder Pain Disability Index)	NR	NR	8

Reddy & Metgud, 2015 [14]	Adhesive capsulitis	30<br>53,4 y	18/12	Pain (Visual Analogue Scale)<br>Range of Motion (Goniometer)<br>Dysfunction (Disabilities of the Arm Shoulder and Hand)	NR	NR	5
Youssef et al, 2015 [15]	Adhesive capsulitis	30<br>54,1 y	NR	Pain (Shoulder Pain Disability Index)<br>Range of Motion (Digital level inclinometer)<br>Dysfunction (Shoulder Pain Disability Index)	NR	NR	5
Haider et al, 2014 [16]	Frozen Shoulder	60<br>46,6 y	17/43	Pain (Visual Analogue Scale)<br>Range of Motion (Goniometer)	NR	NR	5
Arshad et al, 2013 [17]	Frozen Shoulder	100<br>NR	NR	Pain (Visual Analogue Scale)<br>Range of Motion (Goniometer)	NR	NR	5
Doner et al. 2013 [18]	Adhesive capsulitis	40<br>58,9 y	9/31	Pain (Visual Analogue Scale)<br>Range of Motion (Goniometer)<br>Disability (Spanish Shoulder Disability Questionnaire)	NR	NR	5
Patrício et al. 2013 [19]	SIS	26(24)<br>53,8 y	19/5	Pain (Visual Analogue Scale, Neer Shoulder Test, Hawkins-Kennedy Shoulder Test, Algometer)<br>Range of Motion (Postural assessment software / SAPO Version 0.68 software)<br>Disability (Shoulder Pain Disability Index)	NR	9(11,4)	8
Teys et al. 2013 [20]	Shoulder pain	25<br>45,4 y	15/10	Pain (Visual Analogue Scale, Algometer)<br>Range of Motion (Goniometer)	NR	NR	6
Djordjevic et al, 2012 [21]	Painful shoulder	20<br>52,9 y	7/13	Pain (Visual Analogue Scale, Neer and Hawkins-Kennedy Shoulder Tests, Speed Biceps test)<br>Range of Motion (Goniometer)	NR	NR	9

Shrivastava et al. 2011 [22]	Adhesive capsulitis	40<br>59,2 y	19/21	Pain (Visual Analogue Scale)<br>Range of Motion (Goniometer)<br>Disability (Shoulder Pain Disability Index)	NR	NR	4
Kachingwe et al, 2008 [23]	SIS	36(33)<br>46,4 y	17/16	Pain (24-hour Visual Analogue Scale, Neer and Hawkins-Kennedy Shoulder tests)<br>Range of Motion (Goniometer)<br>Disability (Shoulder Pain Disability Index and Functional movement patterns of hand-behind-back or hand-behind-head)	NR	3(9)	8
Teys et al. 2008 [24]	Shoulder pain	24<br>46,1 y	11/13	Pain (Algometer)<br>Range of Motion (Goniometer)	NR	NR	6
Yang et al. 2007 [25]	Frozen Shoulder	28(23)<br>55,6 y	4/24	Range of Motion (Electromagnetic 3-dimensional tracking system - FASTRAK)<br>Disability (FLEX-Short Form)	NR	5(17,8)	8

SIS—Shoulder impingement syndrome; NR—not reported.

Table S3: Summary of findings.

MWM compared to Sham-MWM for [Shoulder]

Patient or population: [Shoulder]
Setting: Rehabilitation
Intervention: MWM
Comparison: Sham-MWM

Outcomes	**Anticipated absolute effects*** (95% CI)		Relative effect (95% CI)	№ of participants (studies)	Certainty of the evidence (GRADE)	Comments
	**Risk with Sham-MWM**	**Risk with MWM**				
Pain	-	SMD **0.63 lower** (1.12 lower to 0.13 lower)	-	66 (2 RCTs)	⊕⊕◯◯ LOW [a,b]	
Shoulder Active Abduction ROM	The mean shoulder Active Abduction ROM was **100.5**	MD **12.66 higher** (1.34 higher to 23.97 higher)	-	90 (3 RCTs)	⊕◯◯◯ VERY LOW [a,b,c]	

* **The risk in the intervention group** (and its 95% confidence interval) is based on the assumed risk in the comparison group and the **relative effect** of the intervention (and its 95% CI).
CI: Confidence interval; **SMD:** Standardised mean difference; **MD:** Mean difference

GRADE Working Group grades of evidence
High certainty: We are very confident that the true effect lies close to that of the estimate of the effect
Moderate certainty: We are moderately confident in the effect estimate: The true effect is likely to be close to the estimate of the effect, but there is a possibility that it is substantially different
Low certainty: Our confidence in the effect estimate is limited: The true effect may be substantially different from the estimate of the effect
Very low certainty: We have very little confidence in the effect estimate: The true effect is likely to be substantially different from the estimate of effect.

Explanations

a. Studies without allocation concealment, random allocation, and/or sample size calculation.

b. Imprecision = total population size less than 400.

c. Meta-analysis with statistical significance in heterogeneity test and high I2.

Table S4: Summary of findings.

MWM+CR compared to CR for [Shoulder]

Patient or population: [Shoulder]
Setting: Rehabilitation
Intervention: MWM+CR
Comparison: CR

Outcomes	**Anticipated absolute effects*** (95% CI)		Relative effect (95% CI)	№ of participants (studies)	Certainty of the evidence (GRADE)	Comments
	**Risk with CR**	**Risk with MWM+CR**				
Pain during activity	The mean pain during activity was **3.3**	MD **2.3 lower** (3.16 lower to 1.44 lower)	-	114 (3 RCTs)	⊕◯◯◯ VERY LOW [a,b,c]	
Pain at rest	The mean pain at rest was **5.4** cm	MD **1.2 cm lower** (2.16 lower to 0.23 lower)	-	100 (3 RCTs)	⊕◯◯◯ VERY LOW [a,b,c]	
Shoulder Active Flexion ROM	The mean shoulder Active Flexion ROM was **27.6 º**	MD **7.11 º higher** (1.85 lower to 16.08 higher)	-	111 (3 RCTs)	⊕◯◯◯ VERY LOW [a,b,c]	
Disability	-	SMD **1.29 lower** (1.89 lower to 0.68 lower)	-	370 (5 RCTs)	⊕◯◯◯ VERY LOW [a,b,c]	
Shoulder Active Abduction ROM	The mean shoulder Active Abduction ROM was **29.3** º	MD **13.47 º higher** (0.86 higher to 26.07 higher)	-	111 (3 RCTs)	⊕◯◯◯ VERY LOW [a,b,c]	
Shoulder Active External Rotation(ER) ROM	The mean shoulder Active External Rotation(ER) ROM was **16.5 º**	MD **4.81 º higher** (0.32 higher to 9.31 higher)	-	111 (3 RCTs)	⊕⊕◯◯ LOW [a,c]	

***The risk in the intervention group** (and its 95% confidence interval) is based on the assumed risk in the comparison group and the **relative effect** of the intervention (and its 95% CI).
CI: Confidence interval; **MD:** Mean difference; **SMD:** Standardised mean difference

**MWM+CR compared to CR for [Shoulder]**						
**Patient or population**: [Shoulder]<br>**Setting**: Rehabilitation<br>**Intervention**: MWM+CR<br>**Comparison**: CR						
Outcomes	**Anticipated absolute effects**[*] (95% CI)		Relative effect (95% CI)	№ of participants (studies)	Certainty of the evidence (GRADE)	Comments
	**Risk with CR**	**Risk with MWM+CR**				
**GRADE Working Group grades of evidence**<br>**High certainty:** We are very confident that the true effect lies close to that of the estimate of the effect<br>**Moderate certainty:** We are moderately confident in the effect estimate: The true effect is likely to be close to the estimate of the effect, but there is a possibility that it is substantially different<br>**Low certainty:** Our confidence in the effect estimate is limited: The true effect may be substantially different from the estimate of the effect<br>**Very low certainty:** We have very little confidence in the effect estimate: The true effect is likely to be substantially different from the estimate of effect.						

Explanations

a. Studies without allocation concealment, random allocation, and/or sample size calculation.
b. Meta-analysis with statistical significance in heterogeneity test and high I2.
c. Imprecision = total population size less than 400

Table S5: Summary of findings.

MWM compared to Maitland for [Shoulder]

Patient or population: [Shoulder]
Setting: Rehabilitation
Intervention: MWM
Comparison: Maitland

Outcomes	**Anticipated absolute effects*** (95% CI)		Relative effect (95% CI)	№ of participants (studies)	Certainty of the evidence (GRADE)	Comments
	**Risk with Maitland**	**Risk with MWM**				
Shoulder Active Abduction ROM	The mean shoulder Active Abduction ROM was **38.9 º**	MD **20.4 º higher** (4.3 higher to 36.5 higher)	-	130 (3 RCTs)	⊕◯◯◯ VERY LOW [a,b,c]	
Shoulder Active Flexion ROM	The mean shoulder Active Flexion ROM was **42.2 º**	MD **22.91 º higher** (1.8 lower to 47.62 higher)	-	130 (3 RCTs)	⊕◯◯◯ VERY LOW [a,b,c]	

***The risk in the intervention group** (and its 95% confidence interval) is based on the assumed risk in the comparison group and the **relative effect** of the intervention (and its 95% CI).
CI: Confidence interval; **MD:** Mean difference

GRADE Working Group grades of evidence
High certainty: We are very confident that the true effect lies close to that of the estimate of the effect
Moderate certainty: We are moderately confident in the effect estimate: The true effect is likely to be close to the estimate of the effect, but there is a possibility that it is substantially different
Low certainty: Our confidence in the effect estimate is limited: The true effect may be substantially different from the estimate of the effect
Very low certainty: We have very little confidence in the effect estimate: The true effect is likely to be substantially different from the estimate of effect.

Explanations

a. Studies without allocation concealment, random allocation, and/or sample size calculation.
b. Meta-analysis with statistical significance in heterogeneity test and high I2.
c. Imprecision = total population size less than 400.

Table S6: Summary of findings.

MWM compared to Exercise for [Shoulder]

Patient or population: [Shoulder]
Setting: Rehabilitation
Intervention: MWM
Comparison: Exercise

Outcomes	**Anticipated absolute effects*** (95% CI)		Relative effect (95% CI)	№ of participants (studies)	Certainty of the evidence (GRADE)	Comments
	**Risk with Exercise**	**Risk with MWM**				
Pain	The mean pain was **1.4** cm	MD **2.62 cm lower** (3.12 lower to 2.12 lower)	-	61 (2 RCTs)	⊕⊕◯◯ LOW [a,b]	

***The risk in the intervention group** (and its 95% confidence interval) is based on the assumed risk in the comparison group and the **relative effect** of the intervention (and its 95% CI).
CI: Confidence interval; **MD:** Mean difference.

GRADE Working Group grades of evidence
High certainty: We are very confident that the true effect lies close to that of the estimate of the effect
Moderate certainty: We are moderately confident in the effect estimate: The true effect is likely to be close to the estimate of the effect, but there is a possibility that it is substantially different
Low certainty: Our confidence in the effect estimate is limited: The true effect may be substantially different from the estimate of the effect
Very low certainty: We have very little confidence in the effect estimate: The true effect is likely to be substantially different from the estimate of effect.

Explanations

a. Studies without allocation concealment, random allocation, and/or sample size calculation.
b. Imprecision = total population size less than 400.

Section and Topic	Item #	Checklist item	Location where item is reported
**TITLE**			
Title	1	Identify the report as a systematic review.	1
**ABSTRACT**			
Abstract	2	See the PRISMA 2020 for Abstracts checklist.	2
**INTRODUCTION**			
Rationale	3	Describe the rationale for the review in the context of existing knowledge.	3
Objectives	4	Provide an explicit statement of the objective(s) or question(s) the review addresses.	4
**METHODS**			
Eligibility criteria	5	Specify the inclusion and exclusion criteria for the review and how studies were grouped for the syntheses.	5
Information sources	6	Specify all databases, registers, websites, organisations, reference lists and other sources searched or consulted to identify studies. Specify the date when each source was last searched or consulted.	5
Search strategy	7	Present the full search strategies for all databases, registers and websites, including any filters and limits used.	5-6
Selection process	8	Specify the methods used to decide whether a study met the inclusion criteria of the review, including how many reviewers screened each record and each report retrieved, whether they worked independently, and if applicable, details of automation tools used in the process.	6
Data collection process	9	Specify the methods used to collect data from reports, including how many reviewers collected data from each report, whether they worked independently, any processes for obtaining or confirming data from study investigators, and if applicable, details of automation tools used in the process.	6
Data items	10a	List and define all outcomes for which data were sought. Specify whether all results that were compatible with each outcome domain in each study were sought (e.g. for all measures, time points, analyses), and if not, the methods used to decide which results to collect.	5-6
	10b	List and define all other variables for which data were sought (e.g. participant and intervention characteristics, funding sources). Describe any assumptions made about any missing or unclear information.	5-6
Study risk of bias assessment	11	Specify the methods used to assess risk of bias in the included studies, including details of the tool(s) used, how many reviewers assessed each study and whether they worked independently, and if applicable, details of automation tools used in the process.	6-7
Effect measures	12	Specify for each outcome the effect measure(s) (e.g. risk ratio, mean difference) used in the synthesis or presentation of results.	7
Synthesis methods	13a	Describe the processes used to decide which studies were eligible for each synthesis (e.g. tabulating the study intervention characteristics and comparing against the planned groups for each synthesis (item #5)).	7-8
	13b	Describe any methods required to prepare the data for presentation or synthesis, such as handling of missing summary statistics, or data conversions.	7-8
	13c	Describe any methods used to tabulate or visually display results of individual studies and syntheses.	8

Section and Topic	Item #	Checklist item	Location where item is reported
	13d	Describe any methods used to synthesize results and provide a rationale for the choice(s). If meta-analysis was performed, describe the model(s), method(s) to identify the presence and extent of statistical heterogeneity, and software package(s) used.	8
	13e	Describe any methods used to explore possible causes of heterogeneity among study results (e.g. subgroup analysis, meta-regression).	8
	13f	Describe any sensitivity analyses conducted to assess robustness of the synthesized results.	8
Reporting bias assessment	14	Describe any methods used to assess risk of bias due to missing results in a synthesis (arising from reporting biases).	8-9
Certainty assessment	15	Describe any methods used to assess certainty (or confidence) in the body of evidence for an outcome.	8-9
**RESULTS**			
Study selection	16a	Describe the results of the search and selection process, from the number of records identified in the search to the number of studies included in the review, ideally using a flow diagram.	9
	16b	Cite studies that might appear to meet the inclusion criteria, but which were excluded, and explain why they were excluded.	9
Study characteristics	17	Cite each included study and present its characteristics.	10
Risk of bias in studies	18	Present assessments of risk of bias for each included study.	10
Results of individual studies	19	For all outcomes, present, for each study: (a) summary statistics for each group (where appropriate) and (b) an effect estimate and its precision (e.g. confidence/credible interval), ideally using structured tables or plots.	10
Results of syntheses	20a	For each synthesis, briefly summarise the characteristics and risk of bias among contributing studies.	10
	20b	Present results of all statistical syntheses conducted. If meta-analysis was done, present for each the summary estimate and its precision (e.g. confidence/credible interval) and measures of statistical heterogeneity. If comparing groups, describe the direction of the effect.	10-13
	20c	Present results of all investigations of possible causes of heterogeneity among study results.	10-15
	20d	Present results of all sensitivity analyses conducted to assess the robustness of the synthesized results.	10-15
Reporting biases	21	Present assessments of risk of bias due to missing results (arising from reporting biases) for each synthesis assessed.	10-15
Certainty of evidence	22	Present assessments of certainty (or confidence) in the body of evidence for each outcome assessed.	10-15
**DISCUSSION**			
Discussion	23a	Provide a general interpretation of the results in the context of other evidence.	16
	23b	Discuss any limitations of the evidence included in the review.	16-17
	23c	Discuss any limitations of the review processes used.	17-18

Section and Topic	Item #	Checklist item	Location where item is reported
	23d	Discuss implications of the results for practice, policy, and future research.	17-18
**OTHER INFORMATION**			
Registration and protocol	24a	Provide registration information for the review, including register name and registration number, or state that the review was not registered.	5
	24b	Indicate where the review protocol can be accessed, or state that a protocol was not prepared.	5
	24c	Describe and explain any amendments to information provided at registration or in the protocol.	NA
Support	25	Describe sources of financial or non-financial support for the review, and the role of the funders or sponsors in the review.	NA
Competing interests	26	Declare any competing interests of review authors.	18-19
Availability of data, code and other materials	27	Report which of the following are publicly available and where they can be found: template data collection forms; data extracted from included studies; data used for all analyses; analytic code; any other materials used in the review.	19

From: Page MJ, McKenzie JE, Bossuyt PM, Boutron I, Hoffmann TC, Mulrow CD, et al. The PRISMA 2020 statement: an updated guideline for reporting systematic reviews. BMJ 2021;372:n71. doi: 10.1136/bmj.n71

For more information, visit: http://www.prisma-statement.org/

References

1. Chandrasekaran, K.; Sundaram, M.; Senthil Selvam, P.; Viswanath Reddy, A.; Senthilkumar, S.; Rathnapandi, V. A comparative study on the effectiveness of Mulligan mobilization versus Positional release therapy technique in patients with Adhesive capsulitis. *Int. J. Res. Pharm. Sci.* **2021**, *12*, 1–5. https://doi.org/10.26452/ijrps.v12i1.3904.
2. Rana, A.A.; Fatima, S.; Sajjad, S.A.; Niaz, M.; Hayat, M.K.; Ahmad, I. Effectiveness of Maitland vs. Mulligan Mobilization Techniques in Adhesive Capsulitis of Shoulder Joint. *Pak. J. Med. Health Sci.* **2021**, *15*, 2561–2564. https://doi.org/10.53350/pjmhs211592561.
3. Fernandes, A.; Shah, L.; Mohan, A. Effectiveness of Kaltenborn Mobilization Technique Versus Mulligan's MWM in Patients with Adhesive Capsulitis of Shoulder. *Indian J. Physiother. Occup. Ther.* **2020**, *14*, 18–24.
4. Subhash, R.; Makhija, M. Effectiveness of Mobilization with Movement in weight bearing position on pain, shoulder range of motion and function in patients with shoulder dysfunction. *Indian J. Public. Health Res. Dev.* **2020**, *11*, 901–905.
5. Ragav, S.; Singh, A. Comparison of Effectiveness of Mulligan 'MWM' Technique versus Kaltenborn Mobilization Technique on Pain and End Range of Motion in Patients with Adhesive Capsulitis of Shoulder Joint: A Randomized Controlled Trial. *J. Exerc. Sci. Physiother.* **2019**, *15*, 1–9.
6. Menek, B.; Tarakci, D.; Algun, Z.C. The effect of Mulligan mobilization on pain and life quality of patients with Rotator cuff syndrome: A randomized controlled trial. *J. Back Musculoskelet. Rehabil.* **2019**, *32*, 171–178. https://doi.org/10.3233/BMR-181230.
7. Rayudu, G.M.; Alagingi, N.K. Efficacy of mulligan technique versus muscle energy technique on Functional ability in subjects with adhesive capsulitis. *Int. J. Recent. Sci. Res.* **2018**, *9*, 25638–25641. https://doi.org/10.24327/IJRSR.
8. Yeole, U.L.; Dighe, P.D.; Gharote, G.M.; Panse, R.S.; Kulkarni, S.A.; Pawar, P.A. Effectiveness of movement with mobilization in adhesive capsulitis of shoulder: Randomized controlled trial. *Indian J. Med. Res. Pharm. Sci.* **2017**, *4*, 1–8.
9. Neelapala, R.; Reddy, R.S.; Danait, R. Effect of Mulligan's posterolateral glide on shoulder rotator strength, scapular upward rotation in shoulder pain subjects—A randomized controlled trial. *J. Musculoskelet. Res.* **2016**, *19*, 1650014.
10. Guimarães, J.F.; Salvini, T.F.; Siqueira, A.L., Jr.; Ribeiro, I.L.; Camargo, P.R.; Alburquerque-Sendín, F. Immediate Effects of Mobilization with Movement vs Sham Technique on Range of Motion, Strength, and Function in Patients with Shoulder Impingement Syndrome: Randomized Clinical Trial. *J. Manip. Physiol. Ther.* **2016**, *39*, 605–615.

11. Delgado-Gil, J.A.; Prado-Robles, E.; Rodrigues-de-Souza, D.P.; Cleland, J.A.; Fernández-de-las-Peñas, C.; Alburquerque-Sendín, F. Effects of mobilization with movement on pain and range of motion in patients with unilateral shoulder impingement syndrome: A randomized controlled trial. *J. Manip. Physiol. Ther.* **2015**, *38*, 245–252.
12. Romero, C.; Torres Lacomba, M.; Castilla Montoro, Y.; Prieto Merino, D.; Pacheco da Costa, S.; Velasco Marchante, M.J. Mobilization with Movement for Shoulder Dysfunction in Older Adults: A Pilot Trial. *J. Chiropr. Med.* **2015**, *14*, 249–258.
13. Satpute, K.H.; Bhandari, P.; Hall, T. Efficacy of hand behind back mobilization with movement for acute shoulder pain and movement impairment: A randomized controlled trial. *J. Manip. Physiol. Ther.* **2015**, *38*, 324–334.
14. Reddy, B.C.; Metgud, S. A randomized controlled trial to investigate the effect of Mlligan's MWM and conventional therapy in stage II Adhesive capsulitis. *Indian. J. Phys. Ther.* **2015**, *3*, 55–59.
15. Youssef, A.R.; Ibrahim, A.M.A.; Ayad, K.E. Mulligan mobilization is more effective in treating diabetic frozen shoulder than the maitland technique. *Int. J. Physiother.* **2015**, *2*, 804–810.
16. Haider, R.; Ahmad, A.; Saum-re-Zahra Hanif, M.K. To compare effects of maitland and mulligan's mobilization techniques in the treatment of frozen shoulder. *Annals* **2014**, *20*, 257–264.
17. Arshad, H.S.; Shah, I.H.; Nasir, R.H. Comparison of Mulligan Mobilization with Movement and End-Range Mobilization Following Maitland Techniques in Patients with Frozen Shoulder in Improving Range of Motion. *Int. J. Health Sci. Res.* **2015**, *4*, 2761–2767.
18. Doner, G.; Guven, Z.; Atalay, A.; Celiker, R. Evaluation of Mulligan's technique for adhesive capsulitis of the shoulder. *J. Rehabil. Med.* **2013**, *45*, 87–91.
19. Patrício, R.I.T. Efeitos Imediatos da Mobilização com Movimento na Dor, Amplitude de Movimento e Actividade Electromiográfica dos Músculos da Cintura Escapular em Indivíduos com Síndrome do Conflito Subacromial. Master's Thesis, Escola Superior de Tecnologia da Saúde do Porto, Instituto Politécnico do Porto, Vila Nova de Gaia, Portugal, 2013.
20. Teys, P.; Bisset, L.; Collins, N.; Coombes, B.; Vicenzino, B. One-week time course of the effects of Mulligan's Mobilisation with Movement and taping in painful shoulders. *Man. Ther.* **2013**, *18*, 372–377.
21. Djordjevic, O.C.; Vukicevic, D.; Katunac, L.; Jovic, S. Mobilization with movement and kinesiotaping compared with a supervised exercise program for painful shoulder: Results of a clinical trial. *J. Manip. Physiol. Ther.* **2012**, *35*, 454–463. https://doi.org/10.1016/j.jmpt.2012.07.006.
22. Shrivastava, A.; Shyam, A.K.; Sabnis, S.; Sancheti, P. Randomized Controlled Study of Mulligan's Vs. Maitland's Mobilization Technique in Adhesive Capsulitis of Shoulder Joint. *Indian J. Physiother. Occup. Ther. Int. J.* **2011**, *5*, 12–15.
23. Kachingwe, A.F.; Phillips, B.; Sletten, E.; Plunkett, S.W. Comparison of manual therapy techniques with therapeutic exercise in the treatment of shoulder impingement: A randomized controlled pilot clinical trial. *J. Man. Manip. Ther.* **2008**, *16*, 238–247.
24. Teys, P.; Bisset, L.; Vicenzino, B. The initial effects of a Mulligan's mobilization with movement technique on range of movement and pressure pain threshold in pain-limited shoulders. *Man. Ther.* **2008**, *13*, 37–42.
25. Yang, J.L.; Chang, C.W.; Chen, S.Y.; Wang, S.F.; Lin, J.J. Mobilization techniques in subjects with frozen shoulder syndrome: Randomized multiple-treatment trial. *Phys. Ther.* **2007**, *87*, 1307–1315.
